# Gene × sex interactions on cognition in the Philadelphia neurodevelopmental cohort

**DOI:** 10.1186/s13293-026-00929-2

**Published:** 2026-06-07

**Authors:** Josephine Mollon, Emma E. M. Knowles, Samuel R. Mathias, Amanda Rodrigue, Ruben C. Gur, Juan Manuel Peralta, Daniel J. Weiner, Elise B. Robinson, Armin Raznahan, Raquel E. Gur, John Blangero, Laura Almasy, David C. Glahn

**Affiliations:** 1 Department of Psychiatry, Boston Children’s Hospital, Harvard Medical School, Boston, MA USA; 2 Brain Behavior Laboratory, Department of Psychiatry, Perelman School of Medicine, Penn-CHOP Lifespan Brain Institute, University of Pennsylvania, Philadelphia, PA USA; 3 School of Medicine, South Texas Diabetes and Obesity Institute, University of Texas of the Rio Grande Valley, Brownsville, TX USA; 4 Analytic and Translational Genetics Unit, Department of Medicine, Massachusetts General Hospital and Harvard Medical School, Boston, MA USA; 5 Stanley Center for Psychiatric Research, Broad Institute of MIT and Harvard, Cambridge, MA USA; 6 Program in Medical and Population Genetics, Broad Institute of MIT and Harvard, Cambridge, MA USA; 7 Section On Developmental Neurogenomics, Human Genetics Branch, National Institute of Mental Health Intramural Research Program, Bethesda, MD USA; 8 Department of Genetics, Perelman School of Medicine, Penn-CHOP Lifespan Brain Institute, University of Pennsylvania, Philadelphia, PA USA; 9 Olin Neuropsychiatry Research Center, Institute of Living, Hartford, CT USA

## Abstract

**Background:**

Small differences between females and males in cognitive abilities have been consistently reported, but the factors underlying these sex differences remain unclear. Social and cultural factors are thought to play a key role, but studies on this topic have been inconclusive. Examination of genetic factors may shed some light on the mechanisms underlying cognitive sex differences.

**Methods:**

Using data from the Philadelphia Neurodevelopmental Cohort, a large, general population sample of individuals aged 8 to 21 years old (N = 4,694), we tested for sex differences in the genetic factors (i.e., Gene × Sex interactions) underlying cognitive ability. Participants completed the Penn Computerized Neurocognitive Battery, which consists of 14 tests designed to capture accuracy and speed in five domains: 1) executive function (abstraction and mental flexibility, attention, working memory), 2) episodic memory (verbal, facial, spatial), 3) complex cognition (verbal reasoning, nonverbal reasoning, spatial processing), 4) social cognition (emotion identification, emotion differentiation, age differentiation), and 5) speed (motor, sensorimotor). Composite domain scores were derived using confirmatory factor analysis, and general accuracy (g) and speed (gs) using principal component analysis.

**Results:**

Small sex differences were observed on most cognitive measures (standardized mean difference (SMD) = 0.061–0.182). Males showed significantly higher genetic variance and lower environmental variance in executive (female σ^2^_g_ = 0.301 v. male σ^2^_g_ = 0.598, p = 0.001, female σ^2^_e_ = 0.243 v. male σ^2^_e_ = 0.024, p = 0.007), and complex (female σ^2^_g_ = 0.291 v. male σ^2^_g_ = 0.610, p = 0.001, female σ^2^_e_ = 0.259 v. male σ^2^_e_ = 0.023, p = 0.006) accuracy. Females showed significantly higher genetic and lower environmental variance on complex (female σ^2^_g_ = 0.575 v. male σ^2^_g_ = 0.135, p = 0.009, female σ^2^_e_ = 0.222 v. male σ^2^_e_ = 0.641, p = 0.012) and social (female σ^2^_g_ = 0.589 v. male σ^2^_g_ = 0.129, p = 0.009, female σ^2^_e_ = 0.236 v. male σ^2^_e_ = 0.672, p = 0.012) speed. Genetic correlations between females and males were not significantly different from 1 on any cognitive measure. Altogether, our results suggest that while the same genetic factors influence cognition in females and males, the magnitude of effect of these genetic factors differs.

**Conclusions:**

We observed small differences between females and males on most cognitive measures, as well as sex differences in heritability on some measures. Future studies are needed to delineate how environmental, genetic, and other biological factors jointly influence cognition.

**Supplementary Information:**

The online version contains supplementary material available at 10.1186/s13293-026-00929-2.

## Plain language summary

Small differences in cognition between females and males have been consistently reported across abilities, cultures, and decades. However, the factors underlying these cognitive sex differences remain unclear. Social and cultural factors are thought to play a key role, but there has been less examination of potential genetic factors. We tested for sex differences in the genetic factors underlying a range of cognitive abilities in a large, general population sample of individuals aged 8 to 21 years old. Small sex differences were observed across most cognitive domains, with female advantages in memory and social cognition, and male advantages in executive and complex cognition. Moreover, differences between females and males in the magnitude of genetic factors underlying cognition were observed for executive, complex, and social cognition, suggesting that some cognitive sex differences are partly driven by sex differences in underlying genetic factors. Most research on the underlying causes of sex differences in cognitive abilities has focused on social and cultural factors, but our findings highlight the importance of considering genetic factors, as well as how these genetic factors act jointly with social and cultural factors to impact cognition. Given the impact of cognition on social, emotional, and health outcomes, further work is needed to delineate the interplay between environmental and genetic factors that underlie cognitive sex differences.

**Supplementary Information:**

The online version contains supplementary material available at 10.1186/s13293-026-00929-2.

## Highlights

Small sex differences in cognitive abilities have been consistently reported, but the factors underlying these differences remain unclear. Social and cultural factors are thought to play a key role, but there has been less examination of potential genetic factors. We tested for sex differences in genetic factors underlying a range of cognitive abilities in a large, general population sample of individuals aged 8 to 21. Small sex differences were observed across most cognitive domains, and sex differences in genetic factors were also observed, suggesting that cognitive sex differences are partly driven by sex differences in genetic factors. While cognitive sex differences are small, they have been consistently reported. Given the impact of cognition on social, emotional, and health outcomes, further work is needed to delineate the interplay between environmental and genetic factors that underlie cognitive sex differences.

**Supplementary Information:**

The online version contains supplementary material available at 10.1186/s13293-026-00929-2.

## Introduction

Whether females and males show differences in cognitive abilities has long been a topic of interest. Towards the end of the twentieth century, research focused on sex differences in mathematics, spatial, and verbal abilities, with multiple meta-analyses conducted on each of these cognitive domains [1]. On the whole, these meta-analyses reported small or negligible differences in abilities between females and males (< 0.2 standard deviations (SD)) [2, 3], except in spatial abilities, where males showed an overall advantage of medium magnitude (0.5–0.7 SD) [4, 5]. The focus on sex differences in mathematics, spatial, and verbal abilities, continued into the twenty-first century [6], compounded by the fact that women continue to be underrepresented in the fields of science, technology, engineering, and mathematics (STEM). However, while a male advantage in spatial abilities [7], and a female advantage in verbal abilities [8, 9] have been found in every single country assessed thus far, findings on sex differences in mathematics and science abilities vary considerably [8, 9].

Much of the research on the underlying causes of sex differences in cognitive abilities has focused on social and cultural factors. These studies have often examined whether 1) sex differences vary across nations, and 2) sex differences correlate with indices of gender equity, with evidence for either taken as support for the importance of social and cultural factors in cognitive sex differences. Overall, these studies have indeed found evidence for variations in cognitive sex differences across, but also within, nations [8–11]. Moreover, certain cognitive sex differences have been found to be correlated with certain indices of gender equity, for example, sex differences in mathematics have been found to be smaller in nations with greater gender equity than in nations with less gender equity [10]. More recently, however, studies have reported no associations between gender equity and sex differences in mathematics [9], as well as larger sex differences in mathematics in more economically developed countries with higher gender equity than in countries with lower gender equity [8]. Similarly, evidence suggests that sex differences in verbal/reading and spatial abilities are correlated with certain, but not all, indices of gender equity, with higher equity being associated with larger differences [9, 11].

More recently, studies have focused on delineating the biological and genetic underpinnings of cognitive sex differences. For example, several studies have reported associations between cognitive ability and testosterone exposure both prenatally and throughout the lifespan, with support for positive, negative, as well as U-shaped associations [12]. To complicate things further, evidence suggests that associations between testosterone and cognitive ability may differ between females and males [13]. Studies on the genetic underpinnings of sex differences have examined differences in genetic liability, i.e., heritability, between females and males. Overall, studies in both general and twin populations have shown that heritability for most complex traits does not significantly differ between females and males [14–16]. Similarly, studies have shown that genetic correlations between females and males for most complex traits are not significantly different from 1, suggesting complete pleiotropy, and therefore, that the same genetic variants underlie these traits in both sexes [17, 18]. On the other hand, studies on rare genetic variants have shown that females carry more large, rare copy number variants (CNVs) with a greater number of affected genes [19, 20]. Moreover, sex-stratified genome-wide association studies (GWAS), albeit on anthropometric traits, have delineated several loci with significant associations in females but not males, and vice versa [21, 22]. Recently, a sex-stratified GWAS of brain anatomy reported largely consistent genetic influences between males and females, with a few exceptions [23].

To date, the genetic underpinnings of cognitive sex differences remain largely unexamined. Using a large, developmental, general population sample, we tested for differences between females and males in the genetic factors underlying cognitive functioning using a novel Gene × Sex (G × S) interaction model. Two different types of Gene × Sex (G × S) interactions were modeled: 1) quantitative G × S interaction tests for differences between females and males in the magnitude of genetic factors, with a significant interaction indicating a difference in magnitude of genetic factors, i.e., heritability, between females and males, and 2) qualitative G × S interaction tests for differences between females and males in the genetic factors themselves, with a significant interaction indicating a difference between females and males in underlying genetic factors. Thus, we tested for differences between females and males in 1) genetic liability i.e., heritability, and 2) genetic factors. We hypothesized that 1) cognitive abilities would be equally heritable in females and males, and 2) the same genetic factors would influence cognition in both sexes.

## Methods

### Participants

The Philadelphia Neurodevelopmental Cohort (PNC) is a population-based sample from the greater Philadelphia area, comprising 9,421 individuals aged 8 to 21 years. The study has been described in detail elsewhere [24]. Participants provided written assent/consent for genomic studies upon providing blood samples during the clinical visit. Inclusion criteria were: 1) ability to provide signed informed consent (assent and parental consent for participants under age 18), 2) English language proficiency, and 3) physical and cognitive ability to participate in computerized cognitive testing. Data are in dbGaP: https://www.ncbi.nlm.nih.gov/projects/gap/cgi-bin/study.cgi?study_id=phs000607.v3.p2. The current analyses were limited to participants who identified as white non-Hispanic (European American), leaving a total of 4,694 subjects with genetic and phenotypic data. Mean age was 13.8 (SD = 3.6) and 50.4% were male (n = 2,366).

### Cognition

All PNC participants completed the Penn Computerized Neurocognitive Battery (CNB) [25–27]. The CNB consists of 14 tests designed to capture functioning in five domains: 1) executive function (abstraction and mental flexibility, attention, working memory), 2) episodic memory (verbal, facial, spatial), 3) complex cognition (verbal reasoning, nonverbal reasoning, spatial processing), 4) social cognition (emotion identification, emotion differentiation, age differentiation), and 5) speed (motor, sensorimotor). Both accuracy and speed (response time) are measured. The CNB also comprises the reading subtest of the Wide Range Achievement Test (WRAT), a measure of general verbal ability.

As in our prior work in this cohort [26, 28, 29], we derived composite domain scores of accuracy and response time in 1) executive function, 2) episodic memory, 3) complex cognition, and 4) social cognition, as well as sensorimotor speed. Confirmatory factor analyses (CFA) were performed in Mplus (version 8) [30] using maximum likelihood (ML) estimation. Factor loadings and fit indices are presented in Supplementary Tables 1 and 2. We also derived composite scores of general accuracy (g), and general speed (gs) as the first component of principal component analyses (PCA) using all accuracy scores, and response times, respectively. To minimize the impact of missing data on these composite scores, the Multivariate Imputation by Chained Equation (MICE) method [31] was used to impute missing values using the mice package in R [32]. The imputation model was based on age, sex and available cognitive data. Test scores were imputed for subjects with less than 50% missing cognitive data. Five datasets were imputed [28] and all subsequent analyses were conducted on imputed data i.e. the average of these five datasets. All cognitive scores were standardized to mean = 0 and SD = 1.

### Genotyping

Samples were genotyped on one of four Illumina arrays: HumanHap550, HumanHap610, OmniExpress or Human1M. Genotyped data were imputed in a separate phase of the study at the Broad Institute [33]. Unobserved genotypes from each chip set were imputed using the IMPUTE2 package and the reference haplotypes in Phase I of the 1000 genomes data (June 2011 release) that included ~ 37,138,905 variants from 1,094 individuals from Africa, Asia, Europe and the Americas. Imputed genotype data were used in subsequent analyses.

### Estimation of genetic relatedness matrix

As detailed previously [28, 29], empirical relatedness was calculated for all pairs of individuals using genotype data. Briefly, 50 k common autosomal SNPs in approximate linkage equilibrium were selected from all available SNP variants after LD pruning (r^2^ > 0.1) using PLINK [34]. Relatedness was then estimated from these SNPs using the IBDLD software package [35](up to 50 SNPs within a 2 cM span).

### Statistical and quantitative genetic analyses

The statistical programming language R [36] was used to generate descriptive statistics and graphics. Genetic analyses were conducted in SOLAR [37] and included all individuals, regardless of relatedness. Since rare variants that may explain a substantial proportion of the phenotypic variance are not well captured by common SNPs, using related individuals is more powerful than using unrelated individuals when estimating heritability. Moreover, using genetically related individuals (even distantly related) is critical for detecting Gene × Sex interactions (see below). When using related individuals, opposite-sex pairs of individuals serve as a design where the same polygenotypes can be observed in both males and females.

*Univariate models.* Briefly, SOLAR implements linear mixed-effects models, which decompose the overall variance of a quantitative trait. Traditionally, these analyses have been performed on family data using matrices calculated from estimated pedigree information, but can also be applied to cohorts of related and unrelated individuals using relatedness estimated from genotype information [38]. Under a univariate polygenic model, the phenotypic variance (σ^2^_p_) of a trait is decomposed into genetic (σ^2^_g_) and environmental (σ^2^_e_) components. Narrow-sense heritability (h^2^) is the proportion of the phenotypic variance accounted for by additive genetic variance (h^2^ = σ^2^_g_/σ^2^_p_). We estimated the heritability of cognitive measures and Gene × Sex (G × S) analyses were then applied to significantly heritable measures.

*Gene* × *Sex (G* × *S) interaction models.* A polygenic model can be extended to examine Gene × Environment (G × E) interactions [39–41]. One potential consequence of a G × E interaction is that the overall additive genetic variance is greater under certain environmental conditions than others. To test for this type of G × E interaction with a discrete environmental variable (e.g. sex), the likelihood of a model in which additive genetic components are allowed to differ between environments (e.g. females and males) is compared with the likelihood of a model in which the additive genetic variances are constrained to be equal. This G × E interaction tests for differences (e.g. between females and males) in action of genetic factors and a significant G × E interaction suggests a difference in magnitude of effect of specific genetic factors. A second potential consequence of a G × E interaction is that the genetic correlation between the trait measured under one environmental condition (e.g. females) and the same trait measured under another environment (e.g. males) is less than 1, implying different genes influencing the trait in the two environments. This phenomenon can be examined where individuals are tested under a single environmental condition, provided the degree of relatedness between individuals is known [41]. To test for this type of G × E interaction, the phenotypic covariance between the trait measured under one environment and the same trait measured under another environment is decomposed into genetic (ρ_g_) and environmental (ρ_e_) components to determine the extent to which the trait is influenced by shared genetic effects in the two environments. To determine whether the genetic correlation (ρ_g_) is significantly less than 1, the likelihood of this model is compared to that of a model where the genetic correlation (ρ_g_) is constrained to 1. Thus, a significant G × E interaction suggests different genes influencing the trait in the two environments (e.g. females and males). Polygenic models with modifications to test for G × E interactions were fitted to all cognitive measures, with sex as the discrete environmental variable i.e., Gene × Sex (G × S) interactions. Importantly, while we consider sex as an environmental variable within this analytic framework, we also recognize that there are important non-environmental aspects to sex.

All models included age, age^2^, sex, and their interactions as fixed-effect covariates. To adjust for multiple testing, the false discovery rate (FDR) was set at 5% [42]. A rank-based inverse normal transformation was applied to all traits to ensure normal distributions.

## Results

A total of 4,694 subjects had available genetic and cognitive data. Mean age of the sample was 13.8 years (SD = 3.6) and 49.6% were female (N = 2,328).

### Cognitive accuracy and speed differ between females and males

**Fig. 1:**
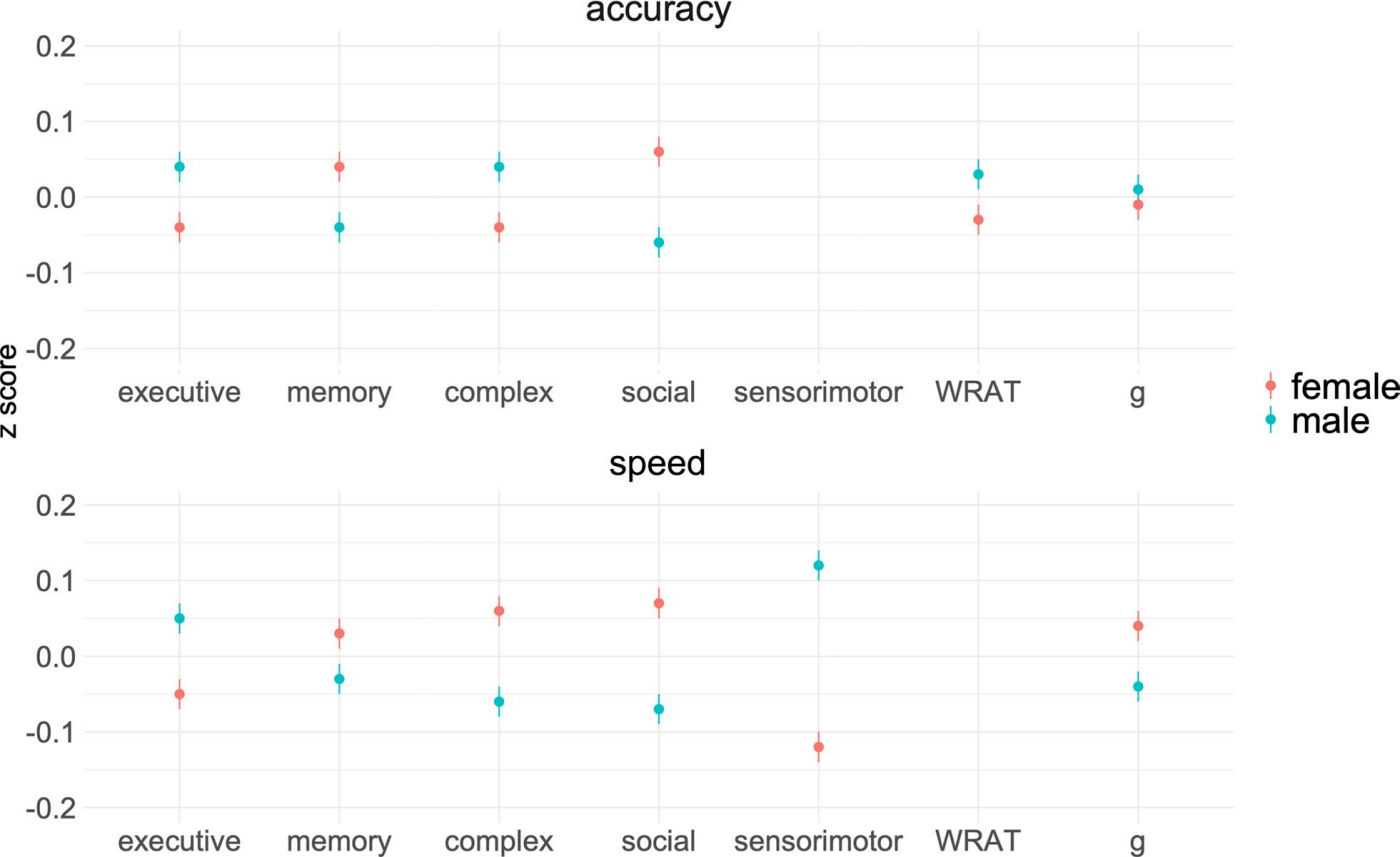
Mean performance on all cognitive measures in females and males

Mean cognitive scores for females and males are presented in Fig. 1, and standardized mean differences (SMD) between females and males are presented in Table 1. As previously reported [25, 27, 43], small, yet statistically significant, differences between females and males were observed on most cognitive measures. Conversely, no significant difference was found between females and males in general accuracy (g) (SMD = 0.020, p = 0.375), although females showed higher general speed (gs) (SMD = 0.073, p = 0.006). Females also showed significantly higher memory (SMD = 0.063, p = 0.013) and social (SMD = 0.102, p = 2 × 10^–5^) accuracy, and faster complex (SMD = 0.118, p = 1 × 10^–5^) and social (SMD = 0.138, p = 5 × 10^–7^) speed. Males showed higher executive (SMD = 0.070, p = 0.002) and complex (SMD = 0.061, p = 0.008) accuracy, and faster executive (SMD = 0.080, p = 0.001) and sensorimotor speed (SMD = 0.182, p = 2 × 10^–16^). All effect sizes were small or negligible (SMD < 0.2)[44].

**Table 1:** Standardized mean difference (SMD), heritability (*h*^2^), genetic (*σ*^2^_g_) and environmental (*σ*^2^_e_) variance, and genetic correlation (*ρ*_g_) for all measures

	Measure	SMD	SE	*p*	*h*^2^	SE	*p*	*σ*^2^_g_			*σ*^2^_e_			*ρ*_g_	SE	*p*
								Female	Male	*p*	Female	Male	*p*			
Accuracy	Executive	**-0.070**	**0.023**	**0.002**	**0.713**	**0.067**	**6 × 10**^**–17**^	**0.301**	**0.598**	**0.001**	**0.243**	**0.024**	**0.007**	1.000	-	-
	Memory	**0.063**	**0.025**	**0.013**	**0.695**	**0.071**	**2 × 10**^**–14**^	0.444	0.654	0.046	0.270	0.072	0.050	0.795	0.195	0.153
	Complex	**-0.061**	**0.023**	**0.008**	**0.696**	**0.069**	**9 × 10**^**–16**^	**0.291**	**0.610**	**0.001**	**0.259**	**0.023**	**0.006**	1.000	-	-
	Social	**0.102**	**0.024**	**2 × 10**^**–5**^	**0.417**	**0.099**	**2 × 10**^**–5**^	0.239	0.338	0.537	0.361	0.329	0.837	0.804	0.429	0.336
	WRAT	-0.048	0.024	0.026	**0.668**	**0.061**	**2 × 10**^**–21**^	0.290	0.438	0.048	0.197	0.124	0.306	1.000	-	-
	General accuracy (*g*)	-0.020	0.027	0.375	**0.718**	**0.072**	**2 × 10**^**–14**^	0.349	0.575	0.021	0.205	0.058	0.099	0.837	0.203	0.215
Speed	Executive	**-0.080**	**0.024**	**0.001**	**0.380**	**0.111**	**4 × 10**^**–4**^	0.312	0.166	0.348	0.318	0.459	0.361	1.000	-	-
	Memory	0.049	0.027	0.068	**0.394**	**0.100**	**3 × 10**^**–5**^	0.442	0.196	0.193	0.352	0.580	0.225	1.000	-	-
	Complex	**0.118**	**0.027**	**1 × 10**^**–5**^	**0.380**	**0.101**	**8 × 10**^**–5**^	**0.575**	**0.135**	**0.009**	**0.222**	**0.641**	**0.012**	1.000	-	-
	Social	**0.138**	**0.027**	**5 × 10**^**–7**^	**0.363**	**0.101**	**1 × 10**^**–4**^	**0.589**	**0.129**	**0.009**	**0.236**	**0.672**	**0.012**	1.000	-	-
	Sensorimotor	**-0.182**	**0.022**	**2 × 10**^**–16**^	**0.471**	**0.090**	**1 × 10**^**–7**^	0.387	0.255	0.219	0.126	0.300	0.099	0.777	0.266	0.246
	General speed (*gs*)	**0.073**	**0.027**	**0.006**	**0.376**	**0.105**	**5 × 10**^**–4**^	0.540	0.137	0.019	0.247	0.626	0.026	1.000	-	-

Bolded estimates signify statistical significance (*p*_FDR_ < .05)

SMD = standardized mean difference between females and males (positive effect sizes signify a female advantage and negative effect sizes signify a male advantage)

*h*^2^ = heritability, *σ*^2^_g_ = genetic variance,* σ*^2^_e_ = environmental variance, *ρ*_g_ = genetic correlation

WRAT = Wide Range Achievement Test

### Cognitive accuracy and speed are heritable

Heritability estimates for all cognitive measures are presented in Table 1. In line with our previous work [28, 29], all accuracy (h^2^ = 0.417–0.718) and speed (h^2^ = 0.363–0.471) measures were moderately and significantly heritable. Since all cognitive measures were heritable, all measures were included in subsequent Gene × Sex (G × S) analyses.

### Heritability of cognitive accuracy and speed differ between females and males

**Fig. 2:**
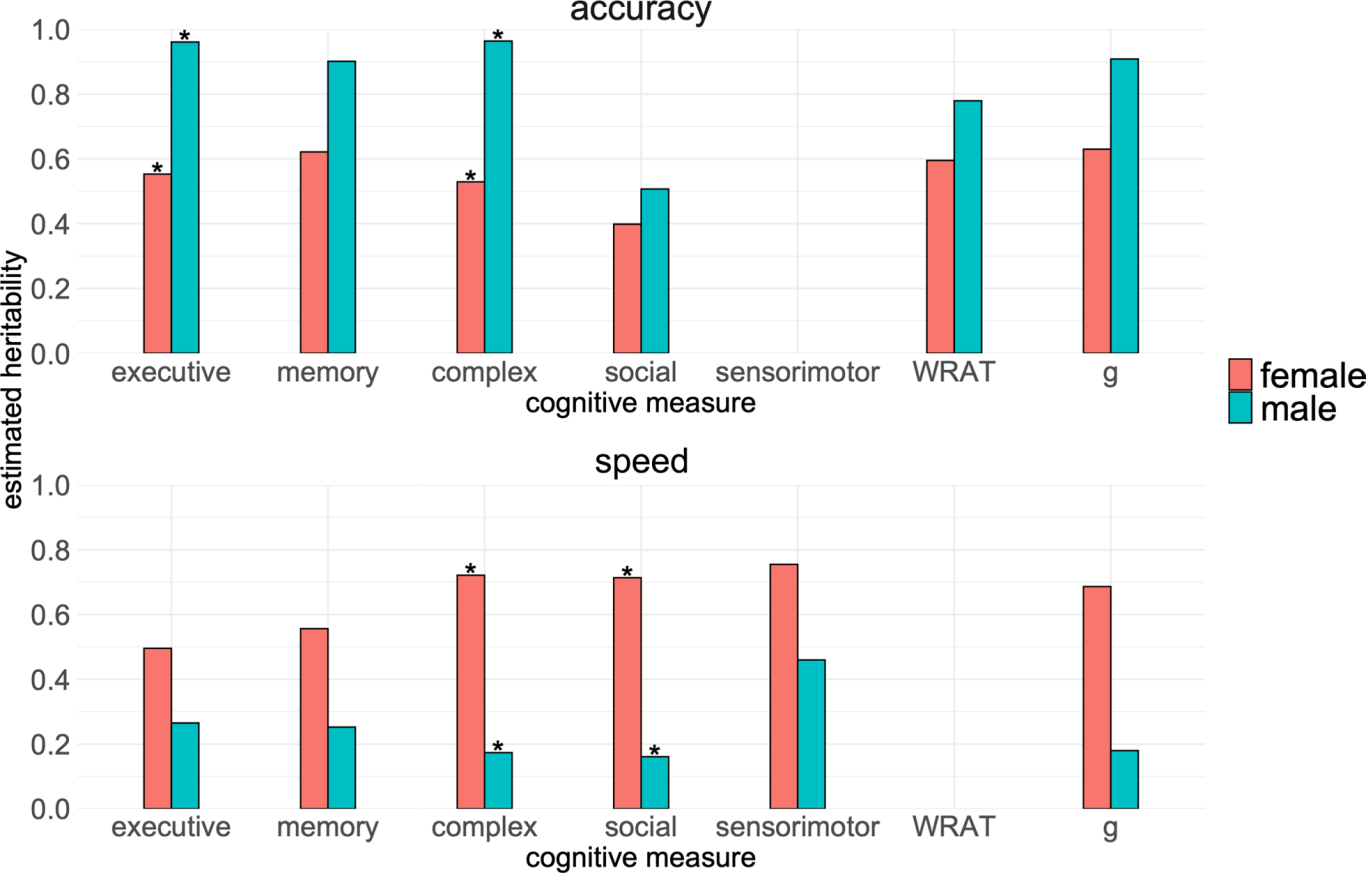
Heritability estimates for all cognitive measures in females and male

Genetic and environmental variance for females and males on all cognitive measures are presented in Table 1 and heritability estimates for females and males are presented in Fig. 2. Males showed significantly higher genetic variance and lower environmental variance in executive (female σ^2^_g_ = 0.301 v. male σ^2^_g_ = 0.598, p = 0.001, female σ^2^_e_ = 0.243 v. male σ^2^_e_ = 0.024, p = 0.007), and complex (female σ^2^_g_ = 0.291 v. male σ^2^_g_ = 0.610, p = 0.001, female σ^2^_e_ = 0.259 v. male σ^2^_e_ = 0.023, p = 0.006) accuracy. Females showed significantly higher genetic and lower environmental variance on complex (female σ^2^_g_ = 0.575 v. male σ^2^_g_ = 0.135, p = 0.009, female σ^2^_e_ = 0.222 v. male σ^2^_e_ = 0.641, p = 0.012) and social speed (female σ^2^_g_ = 0.589 v. male σ^2^_g_ = 0.129, p = 0.009, female σ^2^_e_ = 0.236 v. male σ^2^_e_ = 0.672, p = 0.012). Thus, our results suggest that the magnitude of effect of genetic factors on executive and complex accuracy is higher in males than females, while the magnitude of effect of genetic factors on complex and social speed is higher in females than males.

### Genetic factors underlying cognitive ability do not differ between females and males

Genetic correlations between females and males are presented in Table 1, and were not significantly different from 1 for any cognitive measure. These results suggest that the genetic factors underlying cognitive ability did not significantly differ between females and males, suggesting that the same genetic factors influence cognition in both sexes.

## Discussion

Using a large, developmental, general population cohort, we found small differences between females and males in most cognitive domains, as well as sex differences in the genetic factors underlying some domains. While no sex difference was found in general cognitive accuracy (g), females showed higher general cognitive speed (gs) compared to males. Females also showed higher social and memory accuracy, and faster social and complex speed. Males showed higher executive and complex accuracy, and faster executive and sensorimotor speed. Heritability of social and complex speed was higher in females, while heritability of executive and complex accuracy was higher in males. Conversely, genetic correlations between females and males on all cognitive abilities were not significantly different from 1, suggesting that the same genetic factors influence cognition in both sexes. These novel findings advance prior work on cognitive sex differences, which has largely focused on social and cultural factors, by highlighting the importance of considering sex differences in genetic factors. These findings advance knowledge on the genetic underpinnings of sex differences in cognitive abilities in several ways.

First, we found small differences between females and males across most cognitive domains. Females showed higher social (age differentiation, emotion differentiation, emotion identification) and memory (face, spatial, verbal) accuracy, and faster social and complex (verbal, nonverbal, spatial) speed. Our finding of both higher and faster social cognition in females compared to males is in line with extensive evidence of a female advantage in social cognition [45], particularly in emotion recognition [46–48]. We found a female advantage of small effect size (< 0.2 SD), directly in line with previous meta-analytic evidence [46–48]. Thus, our finding adds to extensive prior evidence of a small, yet robust, female advantage in social cognition, which is consistent across samples and test characteristics [47]. Our findings also add to prior knowledge by showing that differences between females and males in social cognition is partly due to differences between females and males in underlying genetic factors. We found evidence for higher heritability of social cognitive speed in females compared to males, whereby the genetic factors underlying social cognitive speed were amplified in females compared to males, while environmental factors were minimized. Our findings are in line with evidence that cognitive empathy in females, but not males, is predominantly driven by genetic factors [49]. Similarly, a GWAS of cognitive empathy identified a genome-wide significant locus associated with social cognition in females, but not males [50]. Higher heritability in females may be due to gene-environment correlations, whereby females are more likely to evoke or select prosocial experiences based on their underlying genetic predispositions, thus accentuating genetic differences in social cognition [51, 52]. Moreover, genetic factors may be expressed more in females due to maturational and/or hormonal factors [53, 54]. Our findings highlight the importance of taking sex differences into consideration when trying to delineate the genetic underpinnings of social cognition. Moreover, given that social cognitive deficits have been reported in psychiatric disorders [55, 56], and that the prevalence of most psychiatric disorders differs between females and males, future studies aimed at delineating the genetic factors influencing both social cognition and psychiatric risk may provide useful insights into their etiologies.

Second, we found a male advantage in executive (abstraction, attention, working memory) and complex (verbal, nonverbal, spatial) accuracy, as well as executive and sensorimotor speed. Our findings are consistent with an expansive literature on higher spatial abilities in males compared to females [7, 57, 58]. However, we found a male advantage of negligible effect size (< 0.1 SD), while previous studies have reported larger sex differences in spatial ability in cohorts of the same age [57]. This discrepancy is likely because our complex cognitive factor was composed of spatial, but also verbal and nonverbal, reasoning scores. Moreover, the Penn Line Orientation Test (PLOT) measures spatial perception, while a male advantage in spatial abilities has most consistently been reported in tests of mental rotation and manipulation, with smaller sex differences found in spatial perception [59]. Our findings also add to prior knowledge by showing that sex differences in spatial abilities maybe be partly due to sex differences in underlying genetic factors. We found that heritability of complex (verbal, nonverbal, spatial) accuracy was higher in males than females, in contrast with prior evidence. For example, in a study of 4,174 twin pairs, no significant quantitative or qualitative sex differences in genetic and environmental factors on spatial ability were found [60]. Similarly, in a study of 804 twins, heritability of spatial ability did not differ between females and males, although genetic variance was higher in males [61]. In addition to our finding of higher male accuracy and heritability of complex cognition, we also found evidence for higher male accuracy and heritability of executive functions (abstraction, attention, working memory). Prior evidence on sex differences in executive functions shows that results are highly dependent on the tests used. In working memory, for example, females show an advantage in free recall tests, while males show an advantage in complex span tests [62]. Prior evidence on sex differences in genetic factors underlying executive functions is sparse, but a study of 1,294 twins found evidence for differences in genetic and environmental variance between females and males on working memory [63]. Thus, our findings add to limited prior evidence of higher male heritability of executive functions. While higher female heritability has been more consistently reported in social cognition [49, 50], several reasons may account for the sparsity of evidence for higher male heritability. First, higher phenotypic variance in males may lead to higher genetic, but also environmental, variance, and therefore, similar heritability estimates in males and females [61]. Second, higher male heritability may emerge or increase throughout development and, therefore, be age dependent [64]. Nevertheless, our findings of higher heritability in males may reflect gene-environment correlations, whereby males are more likely to evoke or select experiences based on their underlying genetic predispositions, thus accentuating their genetic differences [65, 66]. Moreover, genetic factors may become more expressed in males due to maturational and/or hormonal factors, such as testosterone-driven amplification of genetic factors during puberty [67, 68].

Third, we found high genetic correlations between females and males on all cognitive measures, with none being significantly different from 1, suggesting that the same genetic factors underlie cognition in females and males. Our results are in line with prior evidence from multiple twin studies of no significant qualitative sex differences in the genetic etiology of cognitive ability [1–3]. On the other hand, sex-stratified GWAS have reported genetic correlations between female and male educational attainment that were significantly less than 1, suggesting some heterogeneity between sexes in terms of common genetic variants underlying educational attainment [50, 69]. Similarly, a genome-wide meta-analysis of cognitive empathy reported a genetic correlation between males and females of 0.68 [50]. These discrepant findings are likely due to differences in methodology and power. Even with the powerful twin design, 80% power to detect a genetic correlation of 0.7 (with an estimated heritability of 50%) requires a sample of over 4,000 twins, while detecting a genetic correlation of 0.9 would require a sample of over 30,000 twins [70]. While our sample optimally included both related and unrelated individuals, we still only had 80% power to detect a genetic correlation of 0.7 with an estimated heritability of 70%. Larger studies are needed to test for qualitative sex differences in genetic factors.

This study has limitations. First, our analyses were restricted to European Americans and future studies should include other populations. While our sample also includes individuals of Africa American ancestry, this subgroup is relatively small. Larger samples comprising individuals of African ancestry, as well as Asian, and Hispanic, are needed to establish cognitive sex differences, as well as whether genetic factors underlie these differences. Second, while we used a large sample, and a comprehensive assessment of cognitive abilities, our findings require replication. Our findings demonstrate the utility of composite cognitive scores in genetics studies, since heritability estimates of our composite measures ranged between 36–72%, while heritability estimates of individual cognitive measures were between 8–56% [28, 29]. Nevertheless, replication is needed in larger samples, particularly to detect genetic correlations between females and males that are significantly less than 1, i.e., sex differences in the genetic factors underlying cognition. Finally, while we adjusted for age in our analyses, we were not able to take puberty staging into account. Given the influence of sex hormones on cognition, future studies that are able to take puberty into account are needed. Indeed, future work is needed to delineate other potential physiological and biological (i.e., non-genetic) factors and their influence on cognition.

## Conclusions

Using a large, developmental, general population cohort, we found small differences between females and males in most cognitive domains, as well as sex differences in the genetic factors underlying some domains. We demonstrated quantitative, but not qualitative, sex differences in genetic etiology of cognitive ability, suggesting that while the same genetic factors underlie cognition in females and males, the magnitude of these genetic factors differs between females and males. Our findings advance prior work on cognitive sex differences, which has largely focused on social and cultural factors, by highlighting the importance of considering sex differences in genetic factors, as well as how these genetic factors act jointly with social and cultural factors to impact cognition. Given the impact of cognition on social, emotional, and health outcomes, further work is needed to delineate how environmental and genetic factors jointly influence cognition.

## Supplementary Information

Supplementary file 1.

## Data Availability

PNC data are available in dbGaP: https://www.ncbi.nlm.nih.gov/projects/gap/cgi-bin/study.cgi?study_id=phs000607.v3.p2.

## Declarations

**Ethics approval and consent to participate**

The Philadelphia Neurodevelopmental Cohort (PNC) was approved by the Institutional Review Boards (IRBs) of both the Children’s Hospital of Philadelphia (CHOP) and the University of Pennsylvania.
