# Supplementary material for: Gene × sex interactions on cognition in the Philadelphia neurodevelopmental cohort
Source: Biol Sex Differ. 2026 Jun 7;17:116. doi: 10.1186/s13293-026-00929-2 (PMC13251074; doi:10.1186/s13293-026-00929-2)
Supplement: Supplementary file 1 [file 13293_2026_929_MOESM1_ESM.docx]

| **Supplementary Table 1** Confirmatory factor analyses (CFA) of accuracy scores | | | | |
| --- | --- | --- | --- | --- |
| factor | test | | loading | se |
| executive | abstraction | | 0.434 | 0.014 |
|  | attention | | 0.532 | 0.013 |
|  | working memory | | 0.619 | 0.012 |
| memory | face memory | | 0.705 | 0.014 |
|  | spatial memory | | 0.455 | 0.015 |
|  | verbal memory | | 0.436 | 0.015 |
| complex | verbal reasoning | | 0.785 | 0.008 |
|  | nonverbal reasoning | | 0.683 | 0.009 |
|  | spatial processing | | 0.672 | 0.010 |
| social | age differentiation | | 0.771 | 0.009 |
|  | emotion differentiation | | 0.782 | 0.009 |
|  | emotion identification | | 0.395 | 0.014 |
| Fit indices | | | | |
| RMSEA | | 0.041 | | |
| CFI | | 0.970 | | |
| TLI | | 0.960 | | |

| **Supplementary Table 2** Confirmatory factor analyses (CFA) of reaction times | | | | |
| --- | --- | --- | --- | --- |
| factor | test | | loading | se |
| executive | abstraction | | 0.409 | 0.017 |
|  | attention | | 0.777 | 0.012 |
|  | working memory | | 0.655 | 0.012 |
| memory | face memory | | 0.793 | 0.008 |
|  | spatial memory | | 0.727 | 0.009 |
|  | verbal memory | | 0.781 | 0.008 |
| complex | verbal reasoning | | 0.617 | 0.013 |
|  | nonverbal reasoning | | 0.268 | 0.016 |
|  | spatial processing | | 0.506 | 0.014 |
| social | age differentiation | | 0.666 | 0.011 |
|  | emotion differentiation | | 0.716 | 0.01 |
|  | emotion identification | | 0.711 | 0.009 |
| sensorimotor | sensorimotor speed | | 0.859 | 0.016 |
|  | motor speed | | 0.476 | 0.014 |
| Fit indices | | | | |
| RMSEA | | 0.123 | | |
| CFI | | 0.834 | | |
| TLI | | 0.772 | | |
